# SETD2: from chromatin modifier to multipronged regulator of the genome and beyond

**DOI:** 10.1007/s00018-022-04352-9

**Published:** 2022-06-06

**Authors:** Thom M. Molenaar, Fred van Leeuwen

**Affiliations:** 1 Division of Gene Regulation, Netherlands Cancer Institute, 1066CX Amsterdam, The Netherlands; 2 Department of Medical Biology, Amsterdam UMC Location University of Amsterdam, Meibergdreef 9, 1105AZ Amsterdam, The Netherlands

**Keywords:** SETD2, Set2, H3K36 methylation, Histone methyltransferase, Chromatin, Transcription, Cancer

## Abstract

Histone modifying enzymes play critical roles in many key cellular processes and are appealing proteins for targeting by small molecules in disease. However, while the functions of histone modifying enzymes are often linked to epigenetic regulation of the genome, an emerging theme is that these enzymes often also act by non-catalytic and/or non-epigenetic mechanisms. SETD2 (Set2 in yeast) is best known for associating with the transcription machinery and methylating histone H3 on lysine 36 (H3K36) during transcription. This well-characterized molecular function of SETD2 plays a role in fine-tuning transcription, maintaining chromatin integrity, and mRNA processing. Here we give an overview of the various molecular functions and mechanisms of regulation of H3K36 methylation by Set2/SETD2. These fundamental insights are important to understand SETD2’s role in disease, most notably in cancer in which SETD2 is frequently inactivated. SETD2 also methylates non-histone substrates such as α-tubulin which may promote genome stability and contribute to the tumor-suppressor function of SETD2. Thus, to understand its role in disease, it is important to understand and dissect the multiple roles of SETD2 within the cell. In this review we discuss how histone methylation by Set2/SETD2 has led the way in connecting histone modifications in active regions of the genome to chromatin functions and how SETD2 is leading the way to showing that we also have to look beyond histones to truly understand the physiological role of an ‘epigenetic’ writer enzyme in normal cells and in disease.

## Introduction

All molecular processes in the eukaryotic cell that involve transactions with genomic DNA have to deal with chromatin—the complex of DNA and histone proteins. In general, wrapping DNA around histone octamers to form nucleosomes acts as a barrier for processes that require DNA as a template such as transcription, replication and DNA repair. Indeed, histones need to be removed at least temporarily to allow access to the DNA for these processes (for reviews see [72, 131, 153]). At the same time, nucleosomes also act as an important docking platform for a myriad of factors that regulate DNA transactions. A critical layer of control for these processes is the post-translational modification of histone proteins. Histone post-translational modifications (PTMs) can directly influence the structure of chromatin, for example by neutralizing the positive charge of histone proteins, or act as a docking site for so called chromatin ‘reader’ proteins (for reviews see [24, 141, 176, 223]). Already early on it has been hypothesized that histone PTMs directly control chromatin processes and that a specific combination of histone PTMs can be viewed as a ‘code’ that specifies the function of a DNA region [177]. In the past decade, genome-wide maps for many histone PTMs in different cell types have been generated, which has indeed confirmed that specific histone PTMs often correlate with a DNA element that is in a particular state (i.e. active promoter, active enhancer, site of DNA damage) [10, 174]. However, correlation does not necessarily mean causation. One of the objectives in understanding chromatin is therefore to determine the functional roles of histone PTMs.

A large part of our understanding of chromatin modifying (‘writer’) enzymes comes from research on histone methyltransferases in budding yeast. In *Saccharomyces cerevisiae*, the SET domain-containing protein Set2 methylates histone H3 on lysine 36 (H3K36). This site is located at the base of the N-terminal tail of H3 and can be either mono-, di-, or trimethylated by Set2 [178]. H3K36 methylation was one of the first PTMs for which a clear function was found in transcription. Initial reports found Set2 to be enriched on the coding sequences of active genes suggesting a role in transcription elongation [164, 204], but other functional experiments indicated that Set2 acts as a repressor of transcription [178]. This apparent paradox was solved by the fact that Set2 is indeed associated with actively transcribing gene bodies, but here it represses transcription initiation from so-called ‘cryptic’ promoters through H3K36 methylation thereby maintaining transcriptional fidelity [30, 88, 95, 120]. For other PTMs that were discovered around the same time as H3K36 methylation, namely H3K4 methylation by Set1 and H3K79 methylation by Dot1, a clear functional role in transcription has remained somewhat elusive even till this day [75, 77, 201, 217]. The role of Set2/H3K36 methylation in repressing cryptic transcription is therefore still an important example of how histone methylation PTMs associated with active genes exert their function.

Since Set2’s first function was described in budding yeast, our understanding of Set2 and its homologs in other organisms has greatly expanded. At the molecular level, we are now beginning to get a decent picture of how Set2’s activity towards H3K36 is regulated during transcription. At the same time, the number of functions attributed to Set2 and H3K36 methylation has been steadily growing to include roles in processes such as mRNA splicing and DNA repair. Like with many other chromatin modifiers, Set2 is conserved from yeast to humans, in the form of SETD2 [180]. The apparent ‘canonical’ function of methylating H3K36 during transcription is the same for Set2 and SETD2. However, as is perhaps unsurprising, things are more complicated in multicellular eukaryotes; SETD2 is much larger than its yeast counterpart, SETD2 is but one of several H3K36 methyltransferases, and, excitingly, SETD2 targets not only histone H3 for methylation but non-histone substrates as well—something that has so far not been reported for yeast Set2. This highlights a point that is broadly relevant to chromatin biology; to functionally characterize a particular histone PTM, it is not enough to only interfere with its writer enzyme, for how can one be sure that the observed phenotype is actually attributable to the histone PTM and not to a non-histone substrate or even a non-catalytical function of the writer enzyme? As described in this review, for Set2/SETD2-associated phenotypes there are sometimes discrepancies between different strategies to interfere with Set2/SETD2 function. SETD2 is therefore a prime example of how a true understanding of a chromatin writer enzyme—both at the fundamental level as well as its role in disease—requires teasing apart its various molecular functions and substrates.

## Regulation of Set2/SETD2 activity and substrate recognition

### RNA polymerase II modification during transcription as a key determinant of Set2/SETD2 activity

In both yeast and mammalian cells, the most well-characterized function of Set2/SETD2 is to methylate H3K36 during transcription. This function of Set2/SETD2 is regulated by RNA polymerase II (RNAPII) post-translational modifications. The C-terminal domain of the largest subunit of RNAPII consists of a heptad repeat with the consensus Y_1_S_2_P_3_T_4_S_5_P_6_S_7_. Following the recruitment of RNAPII to the transcription start site, phosphorylation of the serine residues in this repeat stimulates the recruitment of elongation factors and chromatin writers [27, 80]. In general, Serine-5 (Ser5p) phosphorylation is associated with transcription initiation (peaking near 5’ gene ends), while Serine-2 phosphorylation (Ser2p) is linked to transcription elongation (peaking near 3’ gene ends), but both marks can be found along actively transcribed genes (reviewed by [54]). During elongation, RNAPII associates with elongation factors and chromatin writers that simultaneously help the transcription machinery progress through nucleosomes and also promote mRNA processing (for review see [40, 147]). As elongation progresses over the gene body, the combination of RNAPII-CTD Ser5P and Ser2P (denoted as RNAPII-pCTD from hereon) promotes Set2 activity toward H3K36 [113, 164, 178, 204]. Set2-mediated H3K36me3 is therefore predominantly found at the 3’ end of active gene bodies.

### The N-terminal region of Set2 is sufficient for H3K36 methyltransferase activity in vitro

**Fig. 1:**
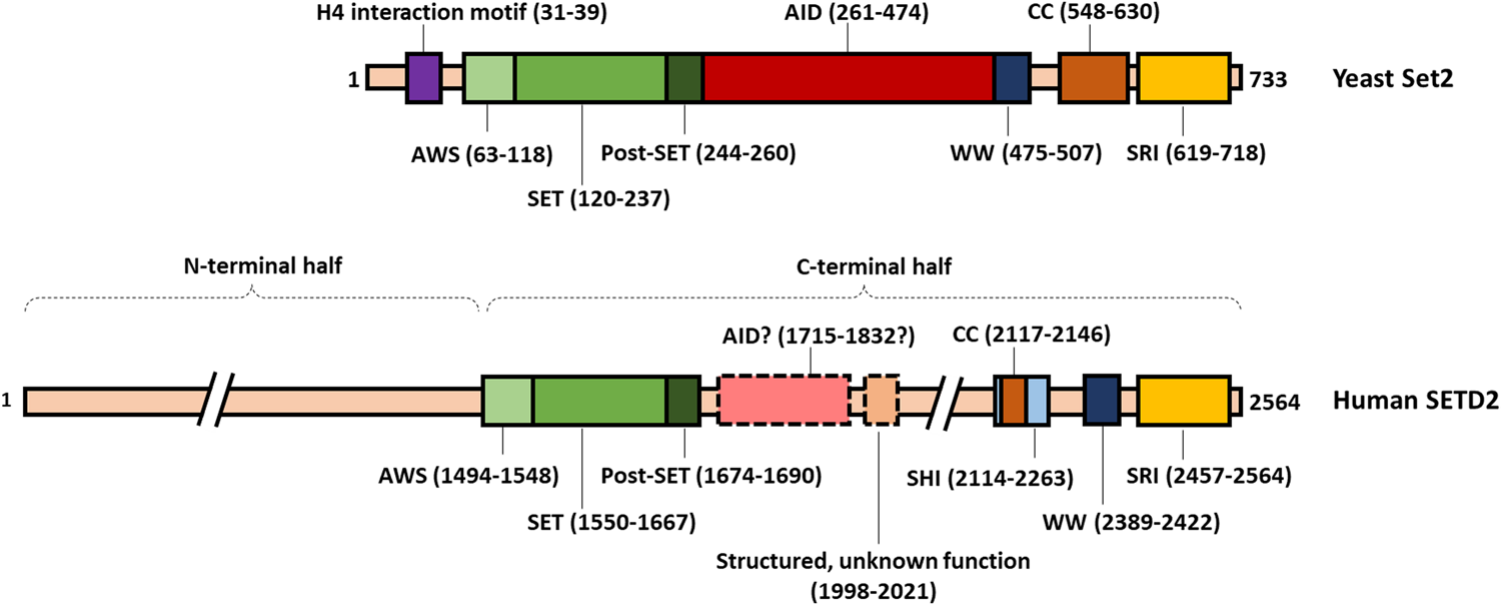
Overview of the protein domains of budding yeast Set2 and human SETD2. *S. cerevisiae* Set2 (733 amino acids) is characterized by a catalytic SET domain flanked by the AWS and post-SET domains, which are also essential for Set2 catalytic activity. The H4-interaction motif at the N-terminus of Set2 contributes to nucleosome interactions via histone H4. The SRI domain stimulates Set2 activity through its direct interaction with RNAPII-pCTD leading to H3K36 trimethylation activity. The structure of the AID, which appears to negatively regulate Set2 activity when the SRI domain is not engaged with RNAPII-pCTD, remains to be experimentally determined. Set2’s human homologue SETD2 (2564 amino acids) has an extended N-terminus which is predicted to be mostly unstructured. Based on structure prediction and sequence homology, the AID is most likely conserved in SETD2, although this remains to be functionally assessed. The SETD2-hnRNP interaction (SHI) domain mediates the interaction between SETD2 and multiple splicing regulators. The SHI domain is not present in budding yeast Set2. *AWS* associated with SET; *AID* auto-inhibition domain; *SRI* Set2-Rbp1 interaction domain; *CC* coiled-coil domain

In *S. cerevisiae,* Set2 is the only H3K36 methyltransferase and is therefore responsible for all H3K36 methylation states [178]. In mammals, multiple enzymes are responsible for H3K36me1 and me2 (for review see [194]). H3K36me3 was thought to be only catalyzed by SETD2 but recently SETD5 (which is a somewhat understudied protein; [186] was shown to catalyze H3K36me3 [169]. In vitro, SETD2 can catalyze all H3K36 methylation states [55] but loss of SETD2 in cells does not affect H3K36me1 and me2 levels indicating that other H3K36 methyltransferases act redundantly for these marks in vivo [52]. All H3K36 methyltransferases identified so far contain a catalytic SET domain which catalyzes the methylation of a lysine residue using S-adenosyl-methionine (SAM) as the methyl donor. In Set2/SETD2, as well as in most other H3K36 methyltransferases, the SET domain is N-terminally flanked by the associated-with-SET (AWS) domain and C-terminally by the post-SET domain (Fig. 1) [194]. These flanking domains together with the SET domain are required for the catalytic activity of Set2 in vitro [98].

Cryo-EM studies show that the AWS-SETD-post-SET domains of yeast Set2 interact with DNA, H3 and the H2A C-terminal tail to help position Set2 on its H3K36 substrate [16, 125]. Human SETD2 interacts with nucleosomes in a similar manner [125]. In yeast, Set2 is further directed to its nucleosomal substrate by a short stretch of acidic amino acids in the N-terminus of Set2 (residues 31–39) that interacts with histone H4 [50]. This H4-interaction motif in Set2 is essential for maintaining normal H3K36me2 and -me3 levels in yeast [50]. Together, the N-terminal region of Set2 encompassing the H4-interaction motif and the AWS-SET-postSET domains (residues 1–261) recapitulate full-length Set2 activity on a nucleosomal substrate in vitro [98, 196]. The Set2 H4-interaction motif has not been identified in human SETD2 so far.

### H2B mono-ubiquitination stimulates Set2/SETD2 activity toward H3K36

A positive regulator of Set2 activity is histone H2B mono-ubiquitination. During transcription elongation, H2B is ubiquitinated at lysine 123 in budding yeast (lysine 120 in humans) by the E2 ubiquitin conjugase Rad6 (UBE2A in humans) and E3 ligase Bre1 (RNF20/40 heterodimer in humans) in a process that also depends on Paf1C [106, 200, 220]. H2Bub stimulates the activity of the H3K4 methyltransferase Set1 and H3K79 methyltransferase Dot1, which is termed *trans* histone cross-talk [47, 84, 182, 188, 192]. H2Bub also stimulates Set2 H3K36 trimethylation activity [16, 191]. Similar as for Set1 and Dot1, H2Bub does not seem to directly promote Set2 recruitment to chromatin. Instead, H2Bub promotes the correct positioning of Set2 on the nucleosome to facilitate H3K36 methylation [16]. In the cryo-EM structure of Set2 with a H2Bub containing nucleosome, the AWS domain also comes into close proximity to ubiquitin providing a potential molecular mechanism for the stimulating effect of H2B mono-ubiquitination on Set2 activity [16]. Consistent with being an elongation mark, H2Bub is enriched over gene bodies in yeast and human cells [193, 199]. H2Bub levels gradually decline towards the 3’ end of gene bodies, particularly in humans [199]. H2Bub levels therefore do not completely overlap with H3K36me3 levels, which peak toward the 3’ end of gene bodies, highlighting that additional factors besides H2Bub such as RNAPII phosphorylation promote full Set2 activity in vivo [156, 168].

### The SRI and AID domains tightly control Set2 H3K36 trimethylation activity

Set2 contains additional domains C-terminally of the catalytic core which control Set2 activity and nucleosomal substrate recognition. These domains are conserved from yeast Set2 to human SETD2. The Set2-Rpb1 interacting (SRI) domain specifically interacts with RNAPII-pCTD [98]. Both the yeast and human SRI domains are characterized by a positive charge, which likely plays a role in the interaction with the negatively charged RNAPII-pCTD. For the human SETD2 SRI domain, it has been shown that a peptide consisting of two RNAPII-CTD heptad repeats with phosphorylated Ser2 and Ser5 interacts as strongly with the SRI domain as peptides with additional phosphorylated repeats [119]. This is consistent with the notion that the RNAPII-pCTD can interact with multiple factors simultaneously during transcription [53]. NMR and functional mutagenesis studies have also identified residues in the human SRI domain that are critical for this interaction, which includes the positively charged residues K2506, R2510 and H2514 as well as the residues V2483 and F2505 [119]. The SRI domain also interacts with DNA, which may contribute to Set2’s preference for nucleosomal substrates over core histones in vitro [196].

**Fig. 2:**
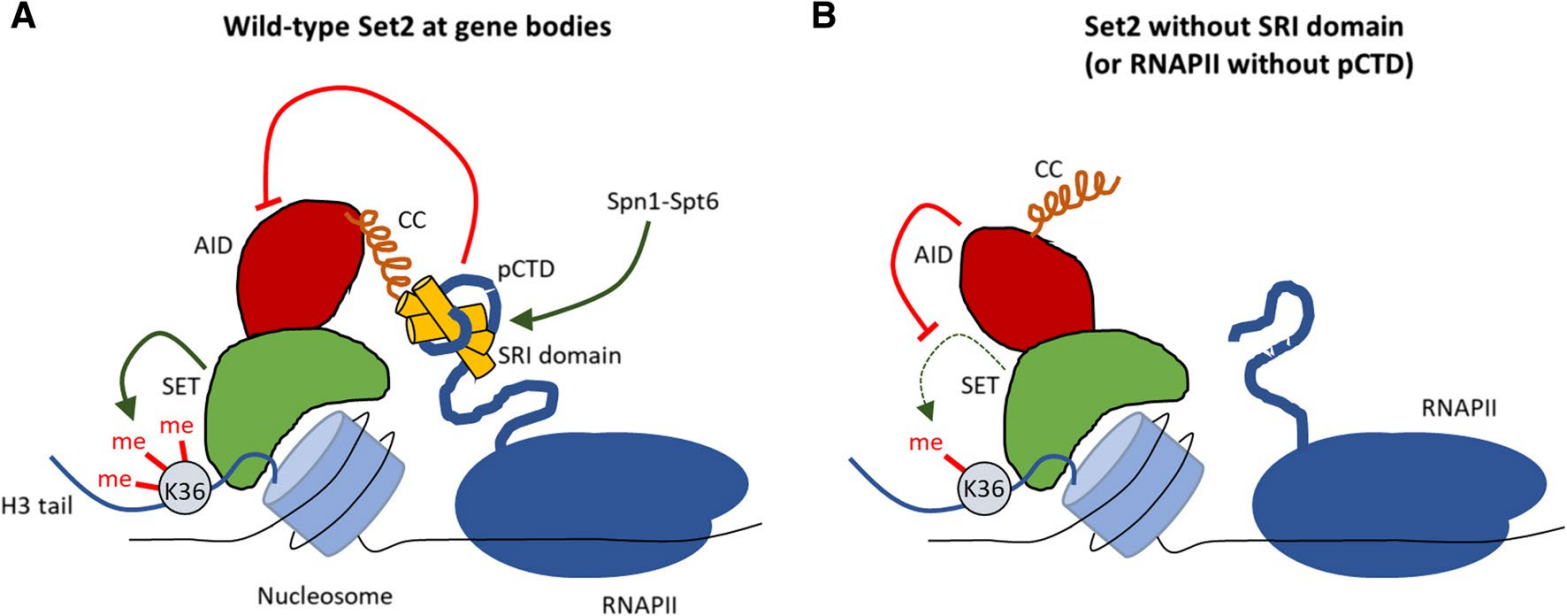
The SRI and AID domain reciprocally regulate Set2 activity. **A** The SRI domain directly interacts with the pCTD of RNAPII. The emerging model is that this interaction not only recruits Set2 to gene bodies but that it also controls Set2 activity by alleviating the negative regulation of the AID on the activity of the SET domain. The Spn1-Spt6 (IWS1-SUPT6H in humans) complex, which directly associates with RNAPII, stimulates the interaction between the SRI domain and the pCTD, although how this works on the molecular level is unknown. **B** In absence of the SRI domain (or in genomic regions where there is no RNA-pCTD) Set2 is unable to trimethylated H3K36

In yeast*,* deletion of the SRI domain (Set2-ΔSRI) abolishes H3K36me3 and reduces H3K36me2 levels [98, 213]. However, Set2-ΔSRI still localizes to chromatin, albeit at reduced levels [66, 213], indicating that the interaction between the SRI domain and RNAPII-pCTD is not essential for the recruitment of Set2 to chromatin. It has therefore been suggested that the SRI-RNAPII-pCTD interaction regulates Set2 activity or substrate recognition rather than strictly localization to the transcription machinery [213]. In support of this hypothesis, fusing Set2 directly to Rpb1 (which is the largest yeast RNAPII subunit and also contains the CTD) does not make the SRI domain dispensable for establishing H3K36me3 [196]. Interestingly, Set2-1-261 and full-length Set2 are more active on nucleosomes in vitro compared to Set2-ΔSRI [196]. Similarly, yeast strains expressing Set2-1-261 have more H3K36me2 (suggesting a higher level of residual activity) than strains expressing Set2-ΔSRI [213]. These findings led to the discovery of an auto-inhibition domain (AID) in the middle of Set2 that regulates Set2 activity as well as protein stability [196]. The SRI domain antagonizes the AID’s negative regulation of the SET domain but the mechanism is not yet understood. The AID does not directly interact with either the SET or SRI domain [66] suggesting that the regulation does not occur via Set2 intramolecular interactions that occlude SET access to its substrate. Regardless of the exact mechanism, the emerging model is that the AID ensures that Set2 only becomes fully active (i.e. becomes competent for H3K36 trimethylation) when Set2 interacts with RNAPII-pCTD through its SRI domain (Fig. 2).

### Elongation factors regulate Set2 activity via the AID-SRI axis

The control of SET domain activity by the AID and SRI domains is influenced by multiple *trans*-acting factors, including the elongation factors Spn1 (IWS1 in humans) and Spt6 (SUPT6H in humans). IWS1/Spn1 has several roles in co-transcriptional processes including pre-mRNA processing and mRNA export from the nucleus [122, 212]. IWS1/Spn1 is directly recruited to the transcription machinery by Spt6 [102, 138], which is a histone chaperone that binds to RNAPII and reassembles nucleosomes in the wake of transcription [23, 40, 85, 147, 211]. Importantly, both IWS1/Spn1 and Spt6 are required to maintain normal H3K36me3 levels in transcribed regions in yeast and human cells [38, 212, 213]. Initially, it was suggested that the mammalian SUPT6H/IWS1 complex promotes H3K36me3 by directly recruiting SETD2, as depletion of IWS1 reduces the interaction between SETD2 and RNAPII in co-immunoprecipitation (co-IP) experiments [212]. However, loss of either Spt6 or Spn1 does not completely abolish Set2 recruitment to chromatin in yeast (as measured by ChIP; [66, 160]. This is reminiscent of how the SRI domain mediates the interaction between Set2 and RNAPII but is not absolutely essential for Set2 localization to chromatin [213]. It is therefore plausible that Spt6-Spn1 controls Set2 activity following its recruitment to chromatin through another mechanism. The observation that Spt6-Spn1 stimulates binding of Set2 to RNAPII strongly suggests that this regulation of Set2 activity by Spt6-Spn1 occurs through the AID-SRI axis. In support of this model, specific point mutations in the AID suppress the reduction in Set2 activity that is associated with Spt6 inactivation in yeast, without significantly altering Set2 recruitment to chromatin [66]. The emerging model therefore is that the Spt6-Spn1 complex stimulates Set2 activity by promoting the association of the SRI domain with RNAPII-pCTD, which in turn relieves the negative regulation by the AID on the catalytic SET domain. Taken together, this tight regulation of Set2 activity during transcription elongation could ensure that H3K36me3 only occurs on active gene bodies.

The Set2 AID appears to be conserved in SETD2 based on the similarity of the predicted structure of the AID in yeast Set2 and human SETD2 using AlphaFold [90]. The predicted structure of the AID resembles that of the transcription factor IIS (TFIIS) N-terminal domain which is a four-helix bundle that is found in a number of chromatin factors, including TFIIS, IWS1/Spn1, MED26 and Elongin A [22, 123]. In most of these factors, the TFIIS N-terminal domain is involved in different protein–protein interactions. In IWS1/Spn1, the TFIIS N-terminal domain mediates the interaction with Spt6 [45]. Although this makes it tempting to speculate that Spt6 directly binds to Set2 through the AID domain, no evidence has been found for a direct interaction between Spt6 and Set2 [66]. Future structural studies of the AID are required to determine if it indeed is part of the TFIIS N-terminal domain family and how it controls the activity of the SET domain. In addition, it will be interesting to determine if the Set2 AID interacts with any proteins and if this interaction influences Set2 activity through the AID-SRI axis.

### SETD2/Set2 WW and CC domain mediate protein–protein interactions

Two other domains in Set2 are the WW domain and the coiled-coil (CC) domain, both of which mediate protein–protein interactions. The WW domain is named for its two conserved tryptophan residues and generally binds to proline-rich proteins. In yeast Set2, deletion of the WW domain does not affect the interaction with RNAPII or H3K36me3 levels [98]. In human SETD2, the WW domain mediates the interaction of SETD2 with a proline-rich region (PRR) in Huntingtin (HTT; [56, 62]. Recently, it has been demonstrated that the SETD2-HTT interaction, together with the actin binding protein HIP1R (HTT-interacting protein 1-related protein), targets SETD2 to trimethylate actin on lysine 68 (ActK68me3) [166]. This modification of actin by the SETD2-HTT-HIP1R complex promotes actin polymerization and cell migration and is an example of a non-histone substrate of SETD2, which will be discussed in more detail below.

The CC domain of SETD2/Set2 was found through in silico prediction as CC domains have a typical amino acid composition. Not much is currently known about the function of the yeast Set2 CC domain. Deletion of the CC domain does not affect yeast Set2 RNAPII binding [98] suggesting that it does not regulate Set2 activity through the AID-SRI axis. CC domains are known to mediate protein homomerization. Interestingly, Set2 has been proposed to occur as a homodimer in cells based on sucrose gradient sedimentation [164, 178]. Human SETD2 also contains a predicted CC that is much shorter than its yeast counterpart. It was recently shown that this CC as well as adjacent unstructured sequences mediate an interaction between SETD2 and heterogeneous nuclear ribonucleoprotein L (hnRNP L), as well as with other mRNA splicing factors [13, 14]. This domain, which has been named the SETD2-hnRNP interaction (SHI) domain, is also important for SETD2’s activity toward H3K36 in vivo [13, 14].

### SETD2 has a large unstructured N-terminal domain

SETD2 has a large N-terminal region (~ 1400 amino acids) that is not present in yeast Set2. This N-terminal region is predicted to be mostly unstructured [15]. Recently, it has been reported that this unstructured domain regulates the stability of SETD2 in a proteasome dependent manner [15]. Both SETD2 and Set2 are highly unstable proteins with a fast turnover rate that is dependent on the proteasome [60]. The N-terminal region of SETD2 contributes to this fast turnover rate [15].

Unstructured domains can also contribute to the liquid–liquid phase separation of proteins. In line with this, SETD2 was recently reported to phase separate in cells [13, 14]. Notably, SETD2’s phase separation behavior is mediated by its C-terminal half which besides the functional domains also contains stretches of intrinsically disordered regions. The large unstructured N-terminal domain does not appear to be directly involved in phase separation of SETD2 [13, 14]. However, it might control liquid droplet formation by regulating the stability of SETD2, as droplet formation is dependent on protein concentration.

## Function of Set2/SETD2 and H3K36 methylation

### H3K36me2/3 promotes histone deacetylation to repress cryptic transcription in budding yeast

**Fig. 3:**
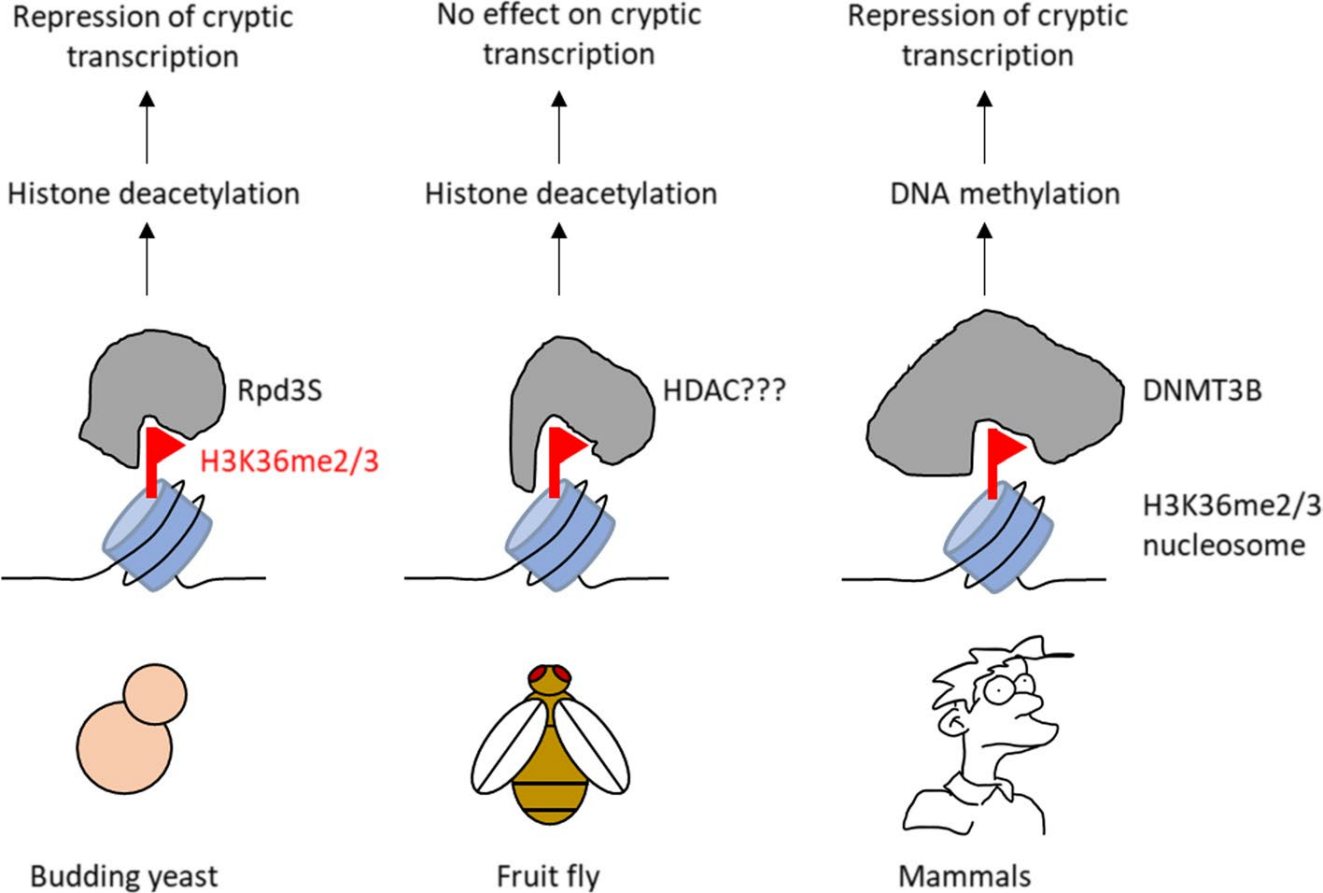
H3K36me2/3 represses cryptic transcription in yeast and human cells by recruiting different repressive chromatin modifiers. In yeast, H3K36me2 and H3K36me3 allosterically activate the Rpd3S HDAC complex to deacetylate chromatin and repress transcription initiation from gene bodies. In Drosophila, H3K36 is dispensable for repression of cryptic transcription. However, H3K36R *Drosophila* cells display increased global levels of H4 acetylation. H3K36me might therefore recruit or activate an HDAC in Drosophila similar as in yeast but this remains to be determined. In human cells, H3K36me3 has been reported to recruit DNMT3B leading the establishment of repressive DNA methylation in active gene bodies which inhibits cryptic transcription

Unlike acetylation, methylation does not change the electric charge of the modified lysine residue, and lysine methylation is therefore believed to mainly function via reader and/or effector proteins. In budding yeast, the most well-characterized function of H3K36me2/3 is to promote chromatin restoration in the wake of transcription by directing the Rpd3S histone deacetylase complex to active gene bodies (Fig. 3) [30, 88, 95, 120, 156, 168]. Whereas transient acetylation of histone tails stimulates elongating RNAPII to progress through nucleosomes (for discussion see [37]), persistent acetylation of gene bodies (hyperacetylation) is believed to lead to cryptic transcription initiation from within coding regions in yeast. To prevent this, the Rpd3S complex is recruited to chromatin by binding to the RNAPII-CTD phosphorylated at Ser5 and Ser2 [49, 67]*.* Following recruitment, activation of Rpd3S requires an interaction between either H3K36me2 or me3 and the Rpd3S subunits Eaf3 and Rco1 [112]. Eaf3 contains a chromodomain that binds H3K36me2/3. This binding is dependent on the plant homeodomain (PHD) of Rco1 which allosterically stimulates recognition of H3K36me2/3 by Eaf3, which in turn stimulates the deacetylation activity of the Rpd3S complex [161]. Rpd3S-mediated deacetylation of histone tails compacts chromatin after transcription elongation and prevents cryptic transcription [30, 95]. Importantly, histone hyperacetylation and cryptic transcription can be observed not only in cells lacking Set2 or Rpd3S subunits but also in yeast cells expressing histone H3 mutated at lysine 36 (H3K36A or H3K36R; [30, 189]. This indicates that chromatin restoration following transcription in budding yeast is indeed mediated by H3K36me and not by either a non-catalytic function or a non-histone substrate of Set2.

Is histone hyperacetylation sufficient to drive cryptic transcription in the absence of the Set2-Rpd3S axis? A general outstanding question is whether histone acetylation directly drives transcription initiation by RNAPII or if histone acetylation is a consequence of transcription. An argument in favor of the first hypothesis is that HATs such as the SAGA and NuA4 complexes are directly recruited to promoters by interacting with transcriptional activators [25, 26, 108]. However, it was recently found that the majority of histone acetylation depends on transcription and that HAT activity (rather than recruitment) requires RNAPII [135]. The same study found that HAT activity on gene bodies also requires transcription. Importantly, histone acetylation not only directly reduces the affinity of histones for DNA but can also promote the recruitment of ATP-dependent chromatin remodeling complexes such as SWI/SNF and RSC through bromodomains that ‘read’ H3/H4ac [5, 34, 89]. These remodeling complexes use ATP to either evict or slide nucleosomes of the promoter to stimulate RNAPII recruitment [159, 185]. Thus, one model could be that in normal cells transcription initiation from cryptic promoters in gene bodies is suppressed by normal nucleosomal spacing and histone deacetylation after each round of transcription. In contrast, failure to deacetylate histones via the Set2-Rpd3S axis could lead to an uncontrolled feedforward loop of chromatin ‘opening’, transcription initiation, and more acetylation in cryptic promoters in gene bodies.

### SETD2 represses cryptic transcription in mammals independently of histone deacetylation

In mammals, SETD2 has also been reported to repress cryptic transcription from within active gene bodies [31, 146]. Notably, yeast Eaf3 is conserved in human cells (where it is called MORF4L1), is a component of the mSIN3A HDAC complex and binds to H3K36me2/3 in vitro similar to its yeast homologue [218]. However, it appears that SETD2 prevents cryptic transcription using a different H3K36me3-mediated mechanism compared to yeast Set2, as H3K36me2 is sufficient to activate Rpd3S in yeast [114] while H3K36me2 levels are not reduced in SETD2 depleted mouse or human cells—at least at the global level [52]. Furthermore, histone deacetylation is generally sufficient to maintain silencing in budding yeast (e.g. at the silent mating type loci and at telomeres) while other eukaryotes have evolved more complex silencing mechanisms that involve chromatin modifications such as H3K9me3, H3K27me3 and/or DNA methylation (all of which are not present in *S. cerevisiae*) in addition to histone deacetylation [187, 190]. In support of the notion that SETD2-mediated repression of cryptic transcription is independent of histone deacetylation, murine SetD2 has been reported to direct CpG island DNA methylation to active gene bodies in mouse embryonic stem cells (mESCs) [146]. Intragenic CpG islands in actively transcribed genes are typically methylated [6] which suppresses cryptic transcription initiation or transcription from (functionally important) alternative intragenic promoters [137]. Importantly, DNA methyltransferase 3B (DNMT3B) has been reported to bind to H3K36me3 through its PWWP reader domain and de novo methylate DNA [7], which suppresses cryptic transcription from gene bodies [146]. Recently, it has also been demonstrated that H3K36me2 recruits and activates DNMT3A through its PWWP domain, which prefers H3K36me2 over H3K36me3 [197, 206]. DNMT3A is therefore targeted to regions enriched in H3K36me2 such as intergenic regions and the most 5’ end of gene bodies [197, 206]. SETD2-mediated H3K36me3 and DNMT3B might therefore have a more prominent role in suppressing transcription from cryptic promoters throughout the gene body compared to HK36me2 and DNMT3A. Mechanistically, it remains to be determined how exactly DNA methylation represses cryptic transcription and whether or not it involves mechanisms such as inhibiting transcription factor binding, recruiting methyl-CpG reader proteins, or cross-talk with other chromatin marks [139],reviewed by [142]. In summary, SETD2 is important for maintaining actively elongating gene bodies in a silenced state in mammalian cells but employs a distinct silencing mechanism compared to budding yeast.

### H3K36 methylation does not repress cryptic transcription in Drosophila

As discussed above, there is substantial evidence that Set2 represses cryptic transcription through H3K36me2/3 in budding yeast, and emerging evidence that SETD2 has a similar function in mammals via H3K36me3. In contrast, Meers et al. [140] reported that H3K36 methylation does not repress cryptic transcription in *Drosophila*. In this landmark study on the function of H3K36 methylation in higher eukaryotes, all the canonical H3 copies were mutated to H3K36R in *Drosophila,* but no evidence could be found for cryptic transcription [140]. So far, this is the only study in metazoans in which the role of H3K36me in cryptic transcription was assessed by directly mutating H3K36 in all canonical H3 copies instead of mutating or depleting SETD2. What causes the discrepancy between the reported roles of H3K36me in mediating repression of cryptic transcription in *Drosophila* and mammalian cells? Unlike most other insects, Diptera (flies) have very low levels of DNA cytosine methylation and lack DNMT1 and DNMT3 [12]. Therefore, if H3K36me3 indeed represses cryptic transcription via DNMT3B recruitment in metazoans, it is perhaps not surprising that this pathway would be absent in *Drosophila.* So far, it has also not been shown that SETD2 represses cryptic transcription in *Drosophila* (only in yeast and mammalian cells)*,* so determining this could be a first step in unraveling this apparent discrepancy. In addition, a challenge for the future is to establish a mammalian model in which all (canonical) H3 copies can be mutated to H3K36R to confirm that SETD2 indeed represses cryptic transcription via H3K36me in mammals, as suggested by Neri et al. [146]. If it turns out that H3K36me3-mediated recruitment of DNMT3B is indeed the major pathway to repress cryptic transcription in human and mouse cells, it will also be interesting to determine how cryptic transcription is repressed in metazoans that have low levels of DNA methylation such as *Drosophila.* Interestingly, H4 acetylation is increased in both H3K36R and SETD2-depleted *Drosophila* cells [9, 140] suggesting that H3K36me might stimulate the activity of an HDAC, similar to Rpd3S in budding yeast. However, this histone hyperacetylation apparently does not cause cryptic transcription in *Drosophila* (Fig. 3), which could be consistent with the notion discussed above that histone deacetylation alone might be sufficient for silencing in budding yeast but not in multicellular eukaryotes*.* It remains to be determined how and where H4 hyperacetylation takes place in *Drosophila* H3K36R cells.

### Set2 represses histone turnover during transcription

In addition to histone hyperacetylation, Set2 deletion leads to increased replication-independent (RI) histone deposition in active gene bodies in budding yeast [189]. Normally, RI histone H3/H4 turnover is high in active promoters and lower in gene bodies [44, 46, 158, 162, 184, 207]. In gene bodies, histone chaperones that associate with elongating RNAPII such as Spt6 and the FACT complex stimulate RNAPII progression through nucleosomes and simultaneously promote nucleosome reassembly after RNAPII passage, thus preventing the need to deposit newly synthesized histones [59, 78, 82, 85]. How does loss of Set2 increase histone turnover in active gene bodies? One possibility is that the hyperacetylation caused by the loss of Set2 directly increases histone turnover rates i.e., by loosening chromatin compaction. To test this hypothesis, it will be interesting to determine if loss of the Rpd3S complex stimulates histone turnover in active gene bodies and if there is genetic interaction with Set2 (i.e., double knockout of Set2 and a Rpd3S subunit such as Rco1). Alternatively, it could be hypothesized that the cryptic transcription initiation itself in gene bodies in *set2* knockout yeast cells is responsible for the increased histone turnover rate. In support of this, it has recently been found in budding yeast that histone turnover in promoter regions is mediated by transcriptional activators [93, 207]. However, because transcriptional activators often directly recruit HATs to promoters, it is difficult to functionally separate transcription initiation from histone acetylation. Interestingly, it has also been shown that H3K9 and H3K14, two lysine residues that are often acetylated in active genes, do not affect histone turnover suggesting that histone acetylation does not directly stimulate histone turnover [58, 93]. However, it could be argued that many residues on H3 and H4 (as well as H2A/H2B) are acetylated during (cryptic) transcription initiation, all of which might contribute to histone turnover. Thus, although there is a clear correlation between histone turnover and acetylation, a causal relationship remains to be established. It is noteworthy to mention that new histones are acetylated at specific sites (e.g. H3K56 in yeast) prior to deposition [136], and as such histone turnover leads to hyperacetylated chromatin [189, 209, 210]. However, it is not yet clear whether the opposite is also true i.e. whether hyperacetylated chromatin directly stimulates histone turnover. Thus, although it is tempting to speculate that hyperacetylation in *set2* knockout yeast cells (or other hyperacetylation conditions) directly leads to a chromatin state in which nucleosomes are constantly lost and replaced, this remains to be functionally assessed.

**Fig. 4:**
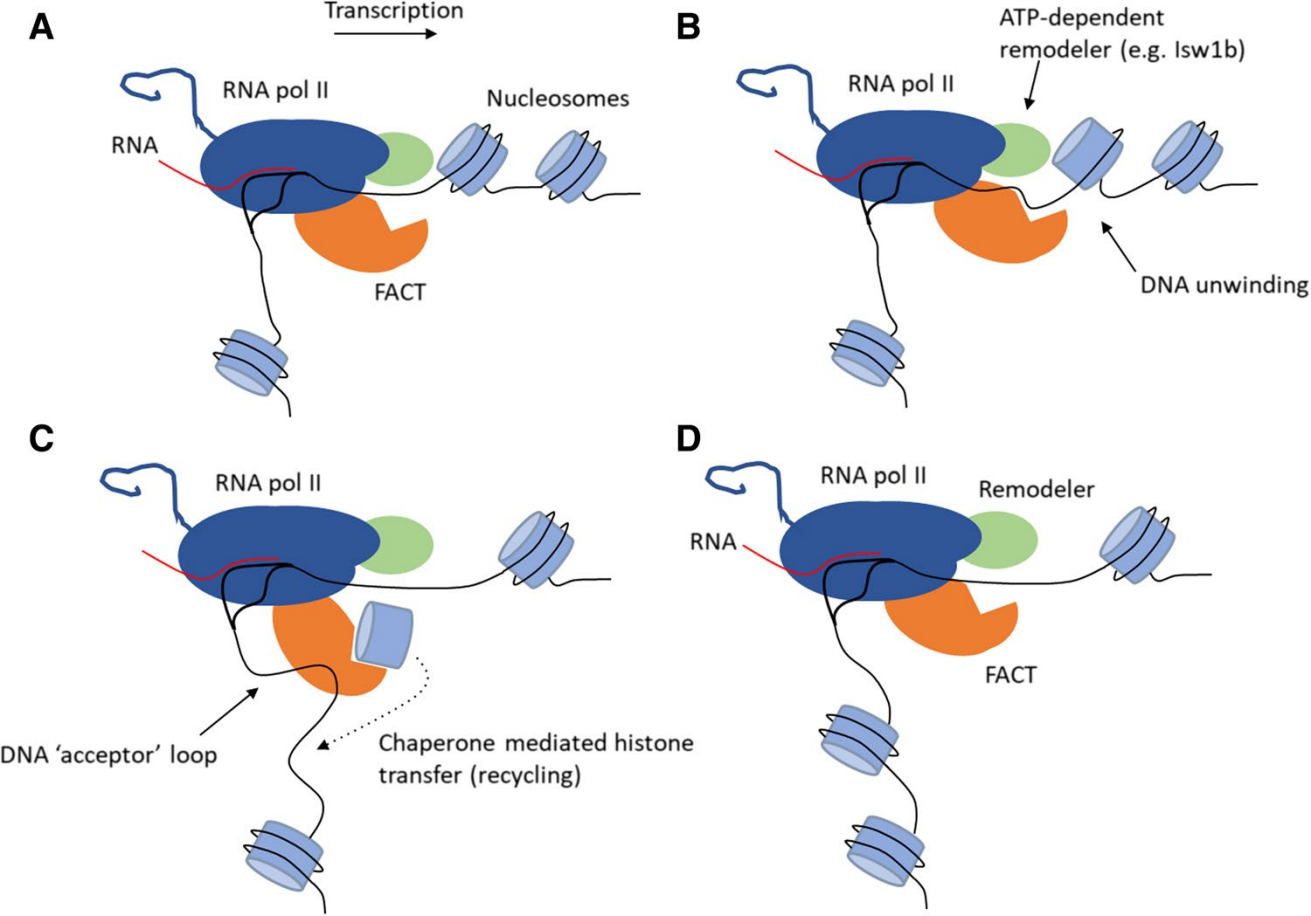
A model for how RNAPII transcribes through chromatin. **A** In vitro, DNA bends about 90° as it exits RNAPII [104, 105]. Two classes of proteins that associate with RNAPII and that are important for transcription through nucleosomes are (i) ATP-dependent remodelers such as the yeast Isw1b complex and (ii) histone chaperones such as the FACT complex. **B** Remodelers use ATP to partially unwind nucleosomes which then become a substrate for FACT. **C** As transcription progresses through the nucleosomal DNA that has been unwound, a naked DNA loop is formed downstream of RNAPII of an estimated size of about 90 bp (including the footprint of RNAPII) [17, 74] that accepts the histone octamer from FACT, which binds to both the H3-H4 tetramer, the H2A-H2B dimers as well as to DNA. The nucleosome is reformed in the wake of transcription. In vitro, the entire histone octamer can be recycled (including H2A-H2B dimers) although this depends on the elongation rate, with H2A-H2B dimers being lost at higher elongation rates [13]. One consequence of the recycling process is that nucleosomes are shifted about 72 bp downstream of their pre-recycling position [13]. Note that this is a model mostly based on in vitro transcription with reconstituted proteins

Set2 might also suppress histone turnover independently of histone acetylation and cryptic transcription initiation. For example, H3K36me3 recruits the chromatin remodeler complex Isw1b [173], which might repress histone turnover by promoting the recycling of resident nucleosomes in the wake of transcription. In addition, H3K36me3 has been reported to inhibit the binding of histone chaperones Asf1, Spt16 (a subunit of the FACT complex) and Spt6 to histone H3, and it has been suggested that this prevents histone turnover [189]. However, it is not exactly clear how this mechanism would work as there is currently no evidence that these chaperones promote histone turnover. As mentioned above, the chaperones Spt6 and FACT promote the reassembly of nucleosomes in the wake of transcription (perhaps in concert with Isw1b, see Fig. 4). This process involves the recycling of old histones and therefore represses histone turnover. In support of this model, FACT has been demonstrated to suppresses the deposition of newly synthesized histone H3 in active gene bodies in budding yeast [82]. FACT also suppresses histone turnover in heterochromatin [145]. It is currently unknown if Set2 represses histone turnover in gene bodies by promoting the chaperoning (and histone recycling) activity of FACT or Spt6. Interestingly, in human cells, loss of SETD2 reduces the occupancy of FACT (but not Spt6) at active gene bodies [31]. It has been suggested that H3K36me3 directly recruits FACT, which is in contrast to the finding that H3K36me3 inhibits yeast Spt16 binding to a histone H3 peptide [189]. To solve this paradox, it will be interesting to determine if Set2 promotes FACT recruitment to chromatin in yeast, and if this mechanism directly involves H3K36me. In addition, because histone PTMs can also allosterically regulate reader proteins rather than directly recruiting them (e.g. Rpd3S activity stimulated by H3K36me2/3), it will be interesting to determine the in vitro histone chaperoning activity of FACT by performing an in vitro transcription assay on a reconstituted chromatin template containing H3K36R nucleosomes. Uncovering how Set2 represses histone turnover and whether this mechanism involves H3K36 methylation promoting histone recycling by chaperones and remodelers like FACT and Isw1b, respectively, will require further studies. It will also be important to determine if Set2-mediated repression of histone turnover in active genes is conserved in mammalian cells.

Lens epithelium-derived growth factor (LEDGF; also known as PSIP1) and hepatoma-derived growth factor 2 (HDGF2) are two H3K36me2/3 reader proteins that have been reported to have FACT-like functions in mammalian cells, namely promoting transcription through a chromatin template [109]. LEDGF binding to chromatin is at least partially dependent on SETD2 [118, 154], suggesting that H3K36me2 (which is not affected by SETD2 loss in mammals) is not completely sufficient for LEDGF recruitment to chromatin. LEDGF and HDGF2 are mainly expressed in differentiated cells, in which FACT expression is low [109], and could therefore fulfill a similar role as FACT in stimulating nucleosome passage by RNAPII and promoting histone recycling during elongation but in distinct cell types. Similar to FACT, it remains to be determined if LEDGF and HDGF2 prevent histone turnover in active gene bodies in mammalian cells.

To summarize, there is evidence in both yeast and human cells that H3K36me3 directly recruits transcription elongation factors that stimulate histone recycling during transcription. This could provide a mechanistic explanation for the observed increase in histone turnover in active genes in absence of Set2 in budding yeast [189]. However, it is noteworthy to mention that so far it has not been definitively proven that Set2-mediated repression of histone turnover is dependent on H3K36me. It has been reported that histone turnover is not altered at several loci in a H3K36R strain, either when the K36R mutation is introduced in resident or in newly synthesized histone H3 [58]. This is an indication that Set2 inhibits histone turnover independently of H3K36me. As a first step to uncovering how exactly Set2 represses histone turnover in budding yeast, it will be important to determine if this function of Set2 depends on its catalytic activity. In addition, Ferrari and Strubin [58] only looked at histone turnover at a limited number of loci in a H3K36R strain. It is theoretically possible that H3K36me represses histone turnover only on a certain class of genes. Therefore, genome-wide analyses of histone turnover rates in H3K36R yeast strains will be useful to determine whether or not H3K36me represses the deposition of newly synthesized histones.

### Set2/SETD2 regulates RNA splicing…

In both yeast and human cells, Set2/SETD2 has been reported to regulate pre-mRNA splicing. In yeast, proper co-transcriptional splicing of several genes has been shown to be dependent on Set2-mediated H3K36me (predominantly H3K36me2) by promoting the recruitment of spliceosome components [175]. This mechanism involves the H3K36me2/3 reader protein Eaf3 (which is also a subunit of Rpd3S) which probably directly recruits splicing factors [110]. In human cells, SETD2 has also been reported to affect mRNA splicing. A genome-wide transcriptome analysis in *SETD2* deficient and proficient primary clear cell renal cell carcinoma (ccRCC) tumors revealed that splicing defects or alternative splicing events such as intron inclusion and differential exon usage are widespread in *SETD2* deficient tumors [171]. Interestingly, in this study it was also reported that *SETD2* deficient cells display increased chromatin accessibility in active genes, which might be due to impaired nucleosome recycling during transcription, as discussed above. The increase in chromatin accessibility may also contribute to the splicing defect observed in *SETD2* deficient cells as nucleosome occupancy is also important for proper mRNA splicing [87].

What evidence is there that SETD2 regulates pre-mRNA splicing via H3K36me3 in human cells? As mentioned previously, the human Eaf3 orthologue Mortality Factor 4 Like 1 (MORF4L1; also known as MRG15) recognizes H3K36me2 and H3K36me3 in vitro [218]. Similar to its role in linking RNA splicing with Set2 activity in yeast, MORF4L1 has been reported to recruit the splicing regulator Polypyrimidine Tract-Binding Protein 1 (PTBP1) to its exon target sites via H3K36me3 to regulate alternative splicing in human cells [130]. Depletion of either MORF4L1 or SETD2 resulted in similar but not completely overlapping splicing defects, suggesting that SETD2 regulates splicing through additional pathways [130]. In the absence of SETD2/H3K36me3, MORF4L1 might still be able to target PTB to exons by binding to H3K36me2, given its in vitro binding properties. However, because H3K36me2 is only enriched at the 5’ end of active gene bodies in humans [57], H3K36me2 is most likely not sufficient to promote PTB localization to the same target exons as H3K36me3.

Another factor that influences splicing through SETD2 activity is Zinc Finger MYND-Type Containing 11 (ZMYND11), which is a chromatin reader protein that has been reported to associate with spliceosome components and regulate intron retention [70]. ZMYND11 binds specifically to the replication-independent ‘gap-filler’ histone variant H3.3 when trimethylated at K36 (H3.3K36me3) through its tandem PHD-, bromo-, and PWWP (PBP) domain, which specifically recognizes both H3K36me3 as well as the S31 residue that is unique to H3.3 compared to canonical H3 [70, 198]. Importantly, splicing regulation by ZMYND11 depends on its chromatin reader domains suggesting that association with H3.3K36me3 is critical for this function of ZMYND11 [70]. This suggests that SETD2-mediated H3K36me3, and specifically H3.3K36me3, regulates splicing via ZMYND11 in human cells. Finally, another line of evidence that SETD2 regulates splicing through H3K36me3 is the finding that the p52 isoform of the H3K36me3 reader LEDGF/PSIP1 binds to multiple splicing regulators such as serine and arginine rich splicing factor 1 (Srsf1) and controls alternative splicing events in mouse cells [157]. LEDGF/PSIP1 might therefore simultaneously regulate both splicing and progression through nucleosomes during transcription elongation [157], [109] making it an important effector of H3K36me3 functions.

### … and splicing also regulates SETD2 activity

There is strong evidence that SETD2 not only regulates splicing but that splicing in turn regulates SETD2. In mammalian cells, H3K36me3 is predominantly found on genes containing introns [2]. This is unlike in yeast, which only has a few intron-containing genes, while most active genes are decorated by H3K36me3. Furthermore, in mammalian cells, depletion of the splicing factor SF3B3 (subunit 3 of the splicing factor 3b protein complex) or treating cells with the SF3B3-targeting drug meayamycin reduces H3K36me3 levels [2]. Along similar lines, inhibiting splicing with Spliceostatin A (which also targets SF3B3) or local removal of splice sites redistributes H3K36me3 levels further towards the 3’ end of genes [97]. Together, this indicates that co-transcriptional splicing regulates SETD2 activity. Thus, splicing globally stimulates H3K36me3 formation by SETD2, and H3K36me3 also regulates local (alternative) splicing events.

As mentioned above, it was recently demonstrated that SETD2 interacts with multiple factors that regulate splicing including hnRNP L through its SHI domain [13, 14]. Do these splicing-regulators recruit SETD2 or does SETD2 recruit these factors to control co-transcriptional splicing? Or is it a combination of both? Depletion of SETD2 affects over one thousand alternative splicing events, of which only a subset is controlled by hnRNP L suggesting that SETD2 is not the sole contributor to hnRNP L function [13, 14]. Since SETD2 interacts with multiple splicing regulators besides hnRNP L, and H3K36me3 itself also regulates splicing, it is currently difficult to untangle the exact relationship between splicing regulation and SETD2.

### H3K36 is not required for splicing in flies

In contrast to yeast and human cells, H3K36me is dispensable for normal splicing in *Drosophila* as no evidence for splicing defects could be found in H3K36R cells [140]. How can H3K36me be important for proper splicing in yeast and human cells, but not in *Drosophila*? In the *Drosophila* study by Meers et al. [140], all canonical H3 copies were replaced by H3K36R but the replication-independent histone variant H3.3 was not mutated. One possibility is therefore that the remaining H3.3K36me3 in these cells is sufficient to promote proper splicing, despite H3.3K36me3 being only a small percentage of the bulk H3K36me3 in wild-type cells. ZMYND11 appears to be conserved in *Drosophila* (but not yeast) based on comparative genomics (Refseq mRNA: NM_136951.3; UniprotKB Q7K264). It is therefore possible that recruitment of this potential ZMYND11 homologue by H3.3K36me3 in H3K36R *Drosophila* cells is sufficient to maintain normal splicing, although this will require further studies. In addition, it will be interesting to determine the role of the *Drosophila* orthologue of Eaf3/MORF4L1 in regulating splicing and if it is independent of H3K36. Furthermore, similar as for cryptic transcription, a mammalian model system in which all H3 copies can be replaced by H3K36R would be a useful tool to directly probe the role of H3K36me in pre-mRNA splicing.

As discussed above, H3K36me is dispensable for repression of cryptic transcription and pre-mRNA splicing in *Drosophila*. However, H3K36R *Drosophila* cells did display a defect in mRNA polyadenylation suggesting a role for H3K36me in mRNA maturation [140]. Messenger RNA polyadenylation is tightly linked to transcription termination. Interestingly, pervasive read-through transcription is a feature of SETD2 mutant ccRCC tumors which also suggests a role for SETD2 in transcription termination in human cells [68]. Notably, a similar role for Set2 in mRNA 3’ end processing and transcription termination has not yet been reported in budding yeast.

### Set2/SETD2 promotes DNA damage signaling and repair

As with other processes that involve DNA transactions, the response to DNA damage is extensively controlled by histones and histone PTMs. Set2/SETD2 has been reported to play multiple roles in the DNA damage response (DDR) in different organisms (for a detailed review see [181]. In budding yeast, Set2 is required for the Mec1/Tel1-mediated phosphorylation of Rad53 and H2AS129 in response to double-strand breaks (DSBs) induced by phleomycin [86]. Rad53ph and H2AS129ph (the yeast equivalent of γH2A.X) are part of the initial signaling cascade induced by DSBs. Impaired Rad53 phosphorylation in response to DSBs has also been observed in H3K36A yeast cells [86] indicating that Set2 promotes DNA damage signaling via H3K36me, although any relevant potential reader protein remains to be found. It was further reported in budding and fission yeast that in response to DSBs, Set2 limits DNA end resection, reduces chromatin accessibility and promotes non-homologous end-joining (NHEJ) over homologous recombination (HR) as the repair pathway for DSBs [86, 148]. This regulation by Set2 occurs at least partially because H3K36me antagonizes H3K36 acetylation (H3K36ac) by Gcn5, which promotes chromatin accessibility leading to increased end resection which in turn favors DSB repair by HR [148]. Set2 has also recently been reported to facilitate transcription coupled-nucleotide excision repair through H3K36 methylation in budding yeast [167] indicating that Set2 regulates multiple DNA repair pathways.

Similar as in yeast, human SETD2 is also involved in the early steps of DDR signaling induced by DNA DSBs. In contrast to Set2 in yeast, however, it appears that in mammalian cells SETD2 promotes DNA repair mainly via HR. SETD2 is required for efficient phosphorylation of ATM as well as p53 (which is an ATM target) but not for γH2A.X formation [32]. SETD2 further promotes DSB repair via HR probably by stimulating the loading of the replication protein A (RPA) ssDNA-binding protein complex and RAD51 on resected DNA ends [32, 92, 154], which is required for the strand-invasion step of HR. Reducing H3K36me3 levels through overexpression of the demethylase KDM4A also leads to HR defects indicating that SETD2 promotes repair through H3K36 methylation [154]. In line with this, the H3K36me2/3 reader LEDGF promotes HR by recruiting the DNA end resection factor C-terminal binding protein interacting protein (CtIP) [43]. In this way, SETD2 provides an important link between active transcription and error-free DSB repair via HR in S/G_2_ (for review see [134]. SETD2 might not exclusively stimulate DSB repair via HR as the H3K36me2/3 reader PHD and Ring finger domains 1 (PHRF1) has been reported to promote NHEJ in human cells [33]. SETD2 has also been reported to stimulate DNA mismatch repair (MMR). Specifically, the MMR protein MSH6 is recruited to chromatin by interacting with H3K36me3 via its PWWP domain [115]. *SETD2* deficient cells display microsatellite instability (MSI) indicating that the interaction between MSH6 and H3K36me3 is important for functional MMR [115]. It is not entirely clear why MMR is selectively targeted to H3K36me3-enriched regions but it could serve to preferentially suppress the accumulation of mutations in actively transcribed genes [81].

### SETD2 promotes S-phase progression

In both budding yeast and human cells, Set2/SETD2 protein levels vary across the cell cycle (being low in G_1_ and peaking in G_2_/M), and Set2 also promotes cell cycle progression by repressing cryptic antisense transcription of cell cycle control genes [48]. Other studies using both fission yeast and human cells have also linked Set2/SETD2 to repressing DNA replication stress. In fission yeast, Set2 is required for efficient origin firing and DNA replication [150]. Mechanistically, Set2 promotes the expression of ribonucleotide reductase (RNR) which is required to maintain normal dNTP levels for efficient replication [149]. Interestingly, SETD2 also regulates dNTP levels in human cells by regulating the expression of the RNR subunit *RRM2* [155]*.* Consistent with this, SETD2 depletion reduces replication fork speed in renal carcinoma cells [92] and deletion of SetD2 leads to replication stress in mouse hematopoietic stem cells [222]. Consequently, *SETD2* deficient cells are sensitive to inhibition of WEE1 which also depletes dNTP levels, leading to S-phase arrest in *SETD2* deficient cells treated with WEE1 inhibitor [155]. This important finding provides an opportunity to selectively target *SETD2* deficient cells, making it a potential much-needed avenue to develop a targeted therapy for cancers in which SETD2 activity is perturbed. Interestingly, it was recently reported that SETD2 also prevents replication stress by methylating histone H3 on lysine 14 (H3K14) [224]. H3K14me3 was found to promote the recruitment of RPA to chromatin leading to ATR activation in response to replication stress. Furthermore, another recent study demonstrated that both budding yeast Set2 and Set1 can methylate histone H3 on lysine 37 (H3K37) to regulate replication origin licensing, and that this mechanism might be conserved in mammals [163]. Thus, Set2/SETD2 regulates normal S-phase progression and there is emerging evidence that this might be in part due to H3 methylation on lysine residues other than H3K36.

### Non-histone substrates of SETD2

Most of the functions of Set2/SETD2 discussed above have been axiomatically linked to Set2/SETD2’s catalytic function towards H3K36. However, an emerging theme in chromatin biology is that many writer enzymes can have functions independent of their catalytic activity, modify non-canonical histone residues (as discussed above), or even modify proteins other than histones [39, 143, 219]. Non-histone substrates have been mostly identified in mammals and only to a lesser extent in yeast, suggesting that writer enzyme functions have diversified in organisms with more complex genomes and proteomes to extend beyond chromatin modification and epigenetic regulation [3]. As such, SETD2 has been reported to methylate numerous non-histone substrates in mammalian cells but so far, no substrates other than H3K36 have been reported for yeast Set2 (though studies systematically looking for non-histone substrates of yeast Set2 are lacking). The first non-H3K36 substrate identified for SETD2 was lysine 40 on the microtubule protein α-tubulin [152]. Trimethylation of α-tubulin (α-TubK40me3) is established by SETD2 during mitosis and cytokinesis and promotes proper chromosome segregation [36, 152]. Microtubule methylation is therefore another way in which SETD2 promotes genome stability, in addition to its role in DNA damage repair. Interestingly, SETD2 mediated α-tubulin methylation was recently found to also occur on the cytoskeleton of post-mitotic neurons indicating that this PTM is not exclusive to the mitotic spindle. Another cytoskeletal protein methylated by SETD2 is actin on lysine 68 (ActK68me3), which promotes actin polymerization and cell migration [166], as discussed above. SETD2 further monomethylates the transcription factor STAT1 on lysine 525 (STAT1-K525me1) which promotes its phosphorylation and activation during the cellular response to viruses [35]. Finally, SETD2 has been shown to monomethylate the catalytic subunit of the Polycomb complex EZH2 at lysine 735 (EZH2-K735me1) which promotes its proteasomal degradation [215]. Given the promiscuity of SETD2, which may be in part due to its tendency to methylate non-structural protein regions (i.e. flexible loops or tails), additional substrates are likely to be discovered.

### The SRI domain controls SETD2 activity toward non-histone substrates

The SRI domain is an important regulator of Set2/SETD2 activity towards H3K36. Recent studies indicate that the SRI domain can also regulate SETD2’s activity towards non-histone substrates. The binding of SETD2 to α-tubulin depends on both the acidic unstructured C-terminal tail of α-tubulin as well as on the SRI domain of SETD2 [94, 152]. Interestingly, a mutation in the SETD2 SRI domain (R2510H) specifically disrupts the interaction between SETD2 and α-tubulin, but not between SETD2 and RNAPII [152]. Importantly, the SETD2-R2510H mutant provides a tool to functionally separate SETD2’s activity towards H3K36 from α-tubulin. Excitingly, neurons of mice heterozygous for Setd2-R2483H (the mouse equivalent of SETD2-R2510H) have defects in axon organization, without any changes in H3K36me3 or gene expression, and these mice also display behavioral changes compared to normal mice [101]. Taken together, these studies indicate that the SRI domain of SETD2 is critical for α-tubulin methylation.

As discussed above, the SRI domain is positively charged at cellular pH and critical positively charged residues facilitate the interaction with the negatively charged RNAPII-pCTD [119]. It is therefore not surprising that the SRI domain of SETD2 also interacts with α-tubulin through its negatively charged C-terminal tail [94]. Interestingly, the AWS-SET-postSET region of SETD2 also directly interacts with α-tubulin in an in vitro setting [152]. One possibility therefore is that the interaction between the SRI domain and α-tubulin is not critical for targeting SETD2 to α-tubulin—as the SET domain might be sufficient for this—but that it controls the activity of the SET domain. This would be analogous to the manner in which the SRI domain controls Set2’s activity towards H3K36 in budding yeast, which also involves the Set2 auto-inhibition domain. It could therefore be determined if mutations in the putative SETD2 auto-inhibition domain can rescue α-TUBK40me3 in cells expressing SETD2-R2510H, similar as to how Set2 AID mutations can by-pass the need for Spt6 in establishing H3K36me3 in yeast [66]. In this scenario, the SRI domain would not only control the activity of the SET domain of SETD2 during the formation of H3K36me3 but also during the methylation of α-tubulin.

Is SRI-dependent methylation of non-H3K36 substrates by SETD2 also conditionally stimulated by post-translational modification of these substrates? The acidic C-terminal tails of both α- and β-tubulin are extensively modified (for review see Janke [83]). Analogous to RNAPII-CTD phosphorylation, certain α-tubulin tail modifications such as phosphorylation and polyglutamylation further increase its negative charge, which could potentially influence its affinity for the positively charged SRI domain. Polyglutamylation—the addition of secondary glutamate chains to glutamate residues—is abundant in the microtubules of neurons [4, 21] as well as in axonemes, the microtubule-based cytoskeleton of cilia and flagella [61, 103, 183]. Given the recent finding that SETD2 methylates cytoskeletal α-tubulin in neurons [101], it will be interesting to determine if α-tubulin C-terminal tail modifications regulate SETD2’s activity in an SRI-dependent manner.

## SETD2’s role in cancer

### SETD2 is a tumor-suppressor gene

Mutations in *SETD2* (both mono- and bi-allelic) have been found in many cancer types, including clear cell renal cell carcinoma (ccRCC), gliomas, and in several types of leukemia [116, 172, 203]. *SETD2* is also mutated in other cancer types such as (among others) lung adenocarcinoma, endometrial carcinoma, colon adenocarcinoma, and melanoma [129], reviewed by [117]. The first cancer-associated *SETD2* mutations were described in ccRCC [42, 51] and much of the subsequent studies have focused on the role of *SETD2* mutations in ccRCC. A frequent mutational event in ccRCC is the (heterozygous) loss of the short arm of chromosome 3 (3p loss; [79], which contains many genes that are also individually mutated in ccRCC (leading to bi-allelic loss) such as *VHL*, *PBRM1*, *BAP1*, and *SETD2*. In ccRCC as well as in other cancer types, truncating mutations occur throughout *SETD2,* and given that the important SET and SRI domains are relatively close to the C-terminus of SETD2, all of these mutations are likely inactivating*.* Missense mutations also occur throughout *SETD2,* although there is a slight clustering of mutations in the catalytic SET domain [29, 42]. For more detailed information about SETD2 mutations in cancer we refer to other resources [41, 129, 172]. The frequent mutation of *SETD2* in multiple types of cancer suggests that *SETD2* is a tumor-suppressor gene. Immunohistochemical stainings of ccRCCs indicate that H3K36me3 is progressively lost from primary tumors to distant metastases [73]. In addition, *SETD2* inactivating mutations are more frequently found in advanced stage ccRCC tumors and correlate with increased tumor recurrence and worse cancer-specific survival [71, 124, 195]. An analysis of the intratumor heterogeneity of several primary ccRCC tumors furthermore revealed that independent *SETD2* mutations tend to arise in distinct sections within a single tumor [63]. Collectively, these studies indicate that (bi-allelic) *SETD2* loss is a relatively late event in ccRCC development and is likely involved in tumor progression rather than initiation [41].

### How does SETD2 loss contribute to tumor progression?

**Fig. 5:**
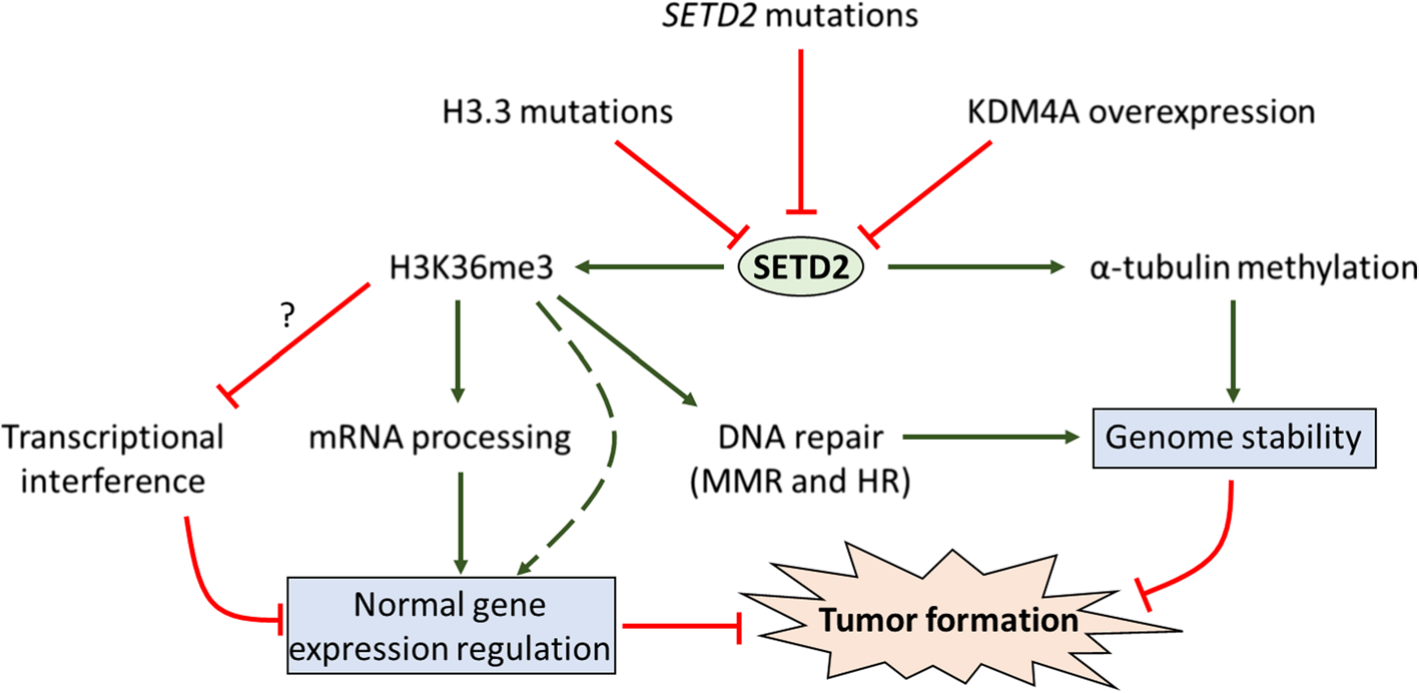
Model for how SETD2 represses tumorigenesis through multiple mechanisms. SETD2’s function can be affected in cancer through direct mutations in the SETD2 gene, or by mutations in H3.3 or deregulated expression of the demethylase KDM4A. SETD2 likely suppresses tumor formation not only through H3K36me3 but also through non-histone substrates such as α-tubulin

So far, SETD2’s tumor-suppressor function has mostly been attributed to its chromatin-associated roles in transcription regulation, mRNA processing and maintaining genome stability (Fig. 5). In ccRCC, loss of SETD2 has been linked to a more accessible chromatin state [171] and enhancer activation which has been suggested to directly promote oncogene expression [205]. As mentioned previously, *SETD2* loss in ccRCC has also been correlated with defects in mRNA splicing, including in known tumor-suppressor genes [171]. In this way, mis-regulated splicing of tumor-suppressor genes in *SETD2* deficient tumors may contribute to tumor progression. In addition, in depth knowledge of splicing regulation by SETD2 (e.g. identifying relevant reader proteins and genes whose splicing is directly affected by SETD2 loss) will be important to uncover the relative contribution of splicing regulation by SETD2 to tumor suppression. In addition to pre-mRNA splicing, SETD2’s function in mRNA polyadenylation and transcription termination has also been suggested to have tumor suppressive effects [68]. In *SETD2* deficient ccRCC cell lines, read-through transcription affects the expression levels of downstream genes and leads to the formation of chimeric transcripts [68]. In budding yeast, read-through transcription either from tandem genes or from antisense transcription generally interferes with the expression of the neighboring gene as transcription elongation passes over the neighboring promoter, leading to chromatin compaction (‘restoration’) via the Rpd3S-Set2 axis [64, 76, 96]. It remains to be determined if a similar transcription interference mechanism occurs in mammalian cells and if it depends on *SETD2,* e.g. through its reported ability to stimulate DNA methylation (as opposed to Rpd3S activation in yeast). Notably, if read-through induced silencing indeed depends on H3K36me3/SETD2 in human cells, then it will be interesting to consider how exactly read-through transcription in *SETD2* deficient cancer cells interferes with downstream genes. Alternative models could include RNAPII collisions or DNA topological stress, for example. A more fundamental insight in the role of SETD2 in transcriptional interference in mammalian cells would greatly contribute to understanding any potential role of this process in tumor progression.

In addition to mRNA processing, SETD2 has tumor suppressor functions through its role in DNA repair (HR and MMR) and promoting chromosome segregation during mitosis (by catalyzing α-TubK40me3). As mentioned previously, the majority of primary ccRCC tumors are characterized by heterozygous 3p loss (leading to loss of one *SETD2* allele)*,* with subsequent mutations in the remaining *SETD2* allele (leading to bi-allelic *SETD2* loss) occurring in more advanced stages of the disease [79]*.* Interestingly, mono-allelic inactivation of *SETD2* does not affect global H3K36me3 levels in ccRCC tumors [73]. This suggests that mono-allelic *SETD2* loss early-on in ccRCC development does not contribute to tumorigenesis via processes that depend on H3K36me3. However, mono-allelic *SETD2* loss does impact α-TubK40me3 levels, leading to mitotic defects and the formation of micronuclei [36, 152]. This indicates that α-tubulin methylation is disproportionally sensitive to loss of one *SETD2* allele (compared to H3K36me3) and that mitotic defects caused by impaired tubulin methylation are likely early drivers in ccRCC tumor development [36]. Subsequent bi-allelic *SETD2* loss due to mutations could further contribute to tumor progression and metastasis by impacting H3K36me3-dependent processes such as MMR (via MSH6) and possibly mRNA processing.

### Oncogenic histone mutations can interfere with SETD2 function

Besides direct *SETD2* loss or mutation, SETD2 function can also be affected in cancer through mutations in histone H3 [28, 91, 99, 126]. H3.3 is a replication-independent variant of H3 that unlike canonical H3 (H3.1 and H3.2) is deposited throughout the cell cycle [1]. In dividing cells, H3.3 is much less abundant than canonical H3 and is enriched in regulatory regions, where it is deposited by a gap-filler mechanism by the H3.3-specific histone cell cycle regulation-defective homologue A (HIRA) complex, and at telomeres, where it is assembled into chromatin by the death domain-associated protein 6 (DAXX)–ATRX complex [28, 65]. A frequent mutation found in chondroblastoma is the substitution of H3.3K36 to methionine (H3.3K36M), with the majority of mutations occurring in *H3F3B* [8]. H3.3K36M mutations also occur at a lower frequency in colorectal cancer [170] and in head and neck squamous cell carcinoma [151]. Even though the mutant H3.3 likely constitutes only a small fraction of the total H3 pool, H3.3K36M leads to a global reduction in H3K36me3 levels [57, 128], suggesting that H3.3K36M inhibits SETD2 in a dominant negative manner (*in trans* inhibition)*.* Structural studies have shown that H3.3K36M rearranges the SET domain, increasing the affinity for the H3 tail and trapping SETD2 on its nucleosomal substrate, thereby explaining how H3.3K36M inhibits SETD2 [209, 210, 221]. H3.3K36M also inhibits the H3K36 dimethyltransferase NSD2 (also known as MMSET) in a manner analogous to SETD2 and therefore also affects global H3K36me2 levels [57].

In chondroblastoma, H3.3K36M has been reported to contribute to tumor development by promoting colony formation, and inhibiting apoptosis and chondrocyte differentiation [57, 128]*.* Interestingly, H3.3K36M also leads to a redistribution of H3K27me3 away from developmentally silenced genes to regions normally enriched in H3K36me3, which may contribute to the derepression of PRC2 target genes that prevent differentiation [128]. In vitro*,* H3K36me3 nucleosomes are a poor substrate for PRC2 [216], providing a possible explanation for this redistribution of H3K27me3 in cells lacking H3K36me3. PRC2-mediated silencing is not strongly impaired in *Drosophila* H3K36R cells [140] so future studies are required to determine if defective PRC2-mediated gene repression is a general feature of cells lacking H3K36me3.

Other mutations that have been found in H3.3 (predominantly in *H3F3A*) are H3.3G34R/V in osteosarcoma [8] and glioblastoma [165, 202], [179], and H3.3G34W/L in giant cell tumor of the bone (GCTB; [8]. Unlike H3.3K36M, these H3.3G34 mutations do not affect global H3K36me3 levels and only inhibit SETD2 *in cis* [111]. H3.3G34 is involved in the binding of SETD2 to H3, fitting in a small pocket in the SET domain, and any substitution with a bulky amino acid residue blocks the interaction [209, 210, 221]. It is currently not clear how H3.3G34 mutations are mechanistically involved in tumor development. A similarity between H3.3K36M and H3.3G34 mutations is that they tend to occur in tumors found in children and young adults. In glioblastoma, H3.3G34R is associated with a developmental expression signature that includes genes that block differentiation (such as self-renewal genes; [18], which is reminiscent of H3.3K36M-mutant chondroblastoma [128]. A common theme might therefore be that H3.3 mutations that affect SETD2 function maintain precursor cells in a pluripotent state and block differentiation during development, although the exact mechanisms behind this process are likely different given that global H3K36me3 levels are unaltered in H3.3G34 mutant cells. In addition, H3.3G34W cells derived from GCTB patients were reported to have mRNA splicing defects which may contribute to tumorigenesis [121]. Expression of H3.3G34R (but not H3.3G34V), as well as H3.3K36M, also impairs DNA repair through HR [127, 154, 208] indicating that genomic instability might be a shared pathway contributing to tumorigenesis in H3.3 mutant and *SETD2* deficient cells.

**Fig. 6:**
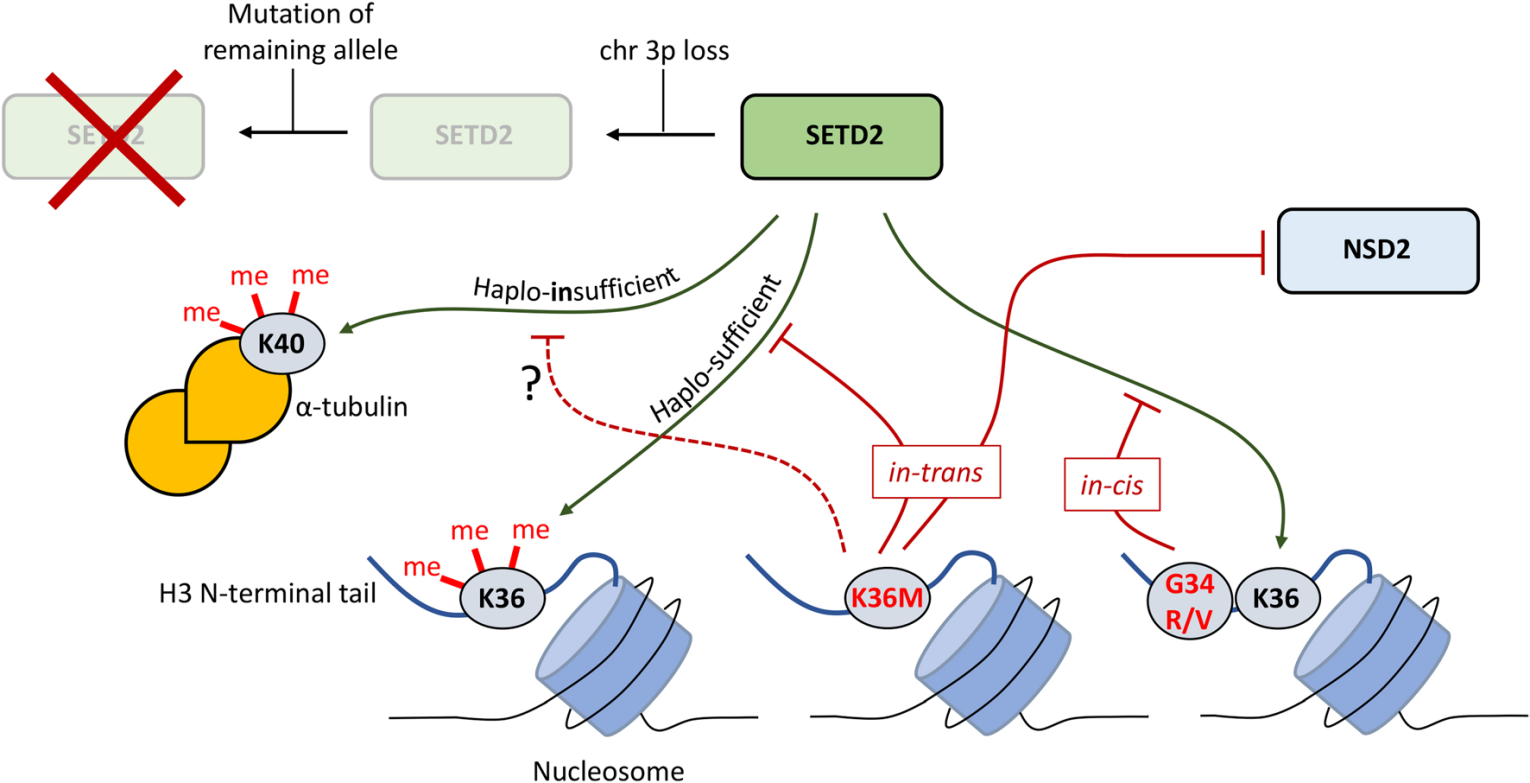
Connection between mutations in SETD2 and histone H3.3 affecting H3K36 methylation. A SETD2 allele can be lost by one-copy deletion of the short arm of chromosome 3, which is a frequent event in ccRCC. Mono-allelic loss of SETD2 does not appear to affect global H3K36me3 levels in ccRCC indicating that SETD2 is haplo-sufficient for H3K36me3. However, α-tubulin methylation on K40 is lost upon mono-allelic SETD2 inactivation suggesting that SETD2-mediated maintenance of genomic stability through tubulin methylation might be frequently perturbed in ccRCC. Mutations in H3.3 (found in chondroblastoma, brain tumors and osteosarcoma among others) can either inhibit SETD2 in-cis (H3.3G34R/V) or in-trans (H3.3K36M). It is currently unknown to what extent H3.3K36M affects the methylation of non-histone substrates of SETD2 such as α-tubulin

To summarize, mutations in H3.3 genes and *SETD2* are found in distinct cancer types and both affect SETD2 activity but can do so in distinct manners (Fig. 6). Besides H3K36me3, SETD2 loss affects the methylation of non-histone substrates of SETD2, whereas H3.3 mutations can also affect other modification states of H3K36 (such as H3K36ac, H3K36me1, and -me2) either by directly preventing the modification or by *in-trans* inhibition of other writers such as NSD2.

### Overexpression of the H3K9me3/H3K36me3-demethylase KDM4A leads to genome instability

SETD2’s function can also be affected in cancer through misregulated expression of the demethylase KDM4A [69], which demethylates both H3K36me3 and H3K9me3 [100]. KDM4A is either deleted or overexpressed (predominantly through gene amplification) in several types of cancer including lung, breast, ovarian, and head and neck cancer [11, 20, 133]. KDM4A promotes S-phase progression and regulates replication timing [19, 20] and its function in cancer is best understood in the context of its overexpression (for a detailed review please see [107, 214]. Interestingly, KDM4A overexpression results in the (extrachromosomal) amplification of chromosome 1q12, through site-specific re-replication during a single cell cycle, and 1q12 amplification also correlates with KDM4A overexpression in tumor samples [20]. Chromosome 1q12 gain mediated by KDM4A overexpression depends on the catalytic activity of KDM4A, and can also be induced by expressing either H3.3K9M or H3.3K36M [20]. This suggests that both H3K36me3 and H3K9me3 prevent 1q12 re-replication during S-phase, although the exact mechanism remains to be determined. KDM4A also has a negative role in DNA repair, inhibiting the recruitment of 53BP1 to DNA damage sites, but this role is independent of its catalytic activity [132] suggesting it is not related to SETD2’s positive role in DNA repair. It is currently unknown if KDM4A further contributes to tumorigenesis by antagonizing SETD2’s role in promoting mRNA processing. Furthermore, it remains to be determined if KDM4A can demethylate non-histone substrates of SETD2 such as α-tubulin, which might be an additional pathway through which KDM4A negatively regulates genome stability. Thus, even though it is clear that both SETD2 loss and KDM4A overexpression perturb H3K36me3 levels, it is not yet entirely clear how much overlap there is in the mechanisms contributing to tumorigenesis in *SETD2* deficient versus KDM4A overexpressing tumors.

## Conclusion

The SETD2/Set2 enzymes have been a prime example of how different approaches in different model systems have led the way in unravelling the molecular role and regulation of a chromatin modifying system associated with RNA polymerase moving along genes. SETD2/Set2 is a key enzyme in the cell involved in a broad range of genome-associated processes. The studies on SETD2/Set2 and H3K36 methylation showcase that teasing apart the various functions requires perturbing not only the writer itself, but also the other domains of the enzymes, the opposing demethylation activity, and the substrate lysine. Moving beyond chromatin, the story of SETD2 emphasizes the importance of knowledge about non-histone substrates of so called ‘epigenetic writers’. An emerging theme in chromatin biology is that non-catalytic functions or activities towards non-histone substrates of epigenetic enzymes need to be considered to fully understand the physiological role of these enzymes. Looking forward, it will be important to develop tools to identify substrates of SETD2 in different cellular contexts in an unbiased way and to perturb SETD2 functions in a substrate-specific manner, e.g. by mutation of a substrate lysine, or by isolation of separation-of-function mutations. With the current advances in genome engineering and proteomics it can be expected that more SETD2 surprises will be discovered and that the function of SETD2 in normal cells and in disease will be further unraveled at a molecular level.

## Data Availability

Not applicable.
